# Acute Laryngitis—II

**Published:** 1910-03-12

**Authors:** 


### Laryngology and Rhinology.

### ACUTE LARYNGITIS—II.

Although simple catarrhal laryngitis is a mild and almost unimportant affection in adults, it presents some special characteristics in children, in whom it is a far more serious malady. The constitutional disturbance is more severe, and is accompanied by rapidity of the pulse and respiration and by a febrile temperature; the latter is usually only moderate in degree and averages about 101° F. There is, however, one feature in particular which should prevent us from considering any case of acute laryngitis in a child as a trivial affection; this is the tendency to dyspnœa, which may come on at any time and with great rapidity. There are two reasons for this: first, the much greater liability in children to spasm of the cords as a reflex result of irritation, and, secondly, the readiness with which œdema ensues on inflammation in a child's larynx.

### Spasmodic Laryngitis.

This is merely catarrhal laryngitis complicated by spasm of the glottis, and is therefore a disease of childhood. The alternative name of it is "laryngitis stridula," and it must be clearly distinguished from laryngismus stridulus, which is a purely spasmodic affection unaccompanied by local disease of the larynx. In the latter the reflex excitability of the nervous system, always active in children, is greatly enhanced by malnutrition; in the former, since a local source of irritation is present, the nervous irritability does not need to be so greatly increased, but we must suppose that it is so to some extent, as all children with catarrhal laryngitis do not get spasm. As a matter of fact a thoroughly healthy child does not usually develop spasmodic laryngitis, and a cause of increased irritability can generally be found in the presence of adenoid hypertrophy and enlargement of the tonsils. The onset is that of an ordinary catarrh affecting the nose and throat, and, in the middle of the night, after a fit of coughing, the spasm comes on. It varies much in severity; in the worst cases there is marked inspiratory stridor, retraction of the epigastrium and lower ribs, and all the signs of asphyxia, which persist until death appears to be imminent, when the spasm relaxes and the child generally goes to sleep. In mild cases there is only a little stridor and distress after coughing. There may be one or more recurrences of the attack during the night, but the symptoms improve in the day. The spasm frequently recurs for a night or two, but with diminishing severity. The affection is never fatal in uncomplicated cases, but is often extremely alarming. It is to be distinguished from œdematous laryngitis by the fact that the dyspnœa completely disappears between the spasms; from laryngeal diphtheria by the same phenomenon, by the general symptoms, and by the fact that the first spasms of laryngitis are the worst; laryngismus stridulus differs in that there is no catarrh, no hoarseness, and indeed no symptom of anything abnormal, between the attacks of spasm. The treatment consists in keeping the child warm in a well-ventilated room, the use of a steam kettle, and the exhibition of ipecacuanha, combined, if desired, with antimony. The ipecacuanha should first be given in doses sufficient to produce emesis, and then continued in smaller quantity as an expectorant. The attacks of spasm are short and sharp, and there is no time to put the child into a hot bath, a recommendation one occasionally sees; but the inhalation of amyl nitrite from a capsule may be tried.

### Œdematous Laryngitis.

In children this is an occasional result of catarrhal inflammation; it may affect the upper aperture or the subglottic region, and is apt to arise with alarming rapidity. Dysphagia is rarely noticed in children, and dyspnœa is the principal symptom. Like all acute laryngeal affections in children it is apt to be complicated by spasm, but between the spasms the dyspnœa does not entirely subside. The onset is more sudden than that of diphtheria, in which there is nearly always a history of previous malaise. The treatment is the same as that of spasmodic laryngitis, but, if the dyspnœa fails to respond to treatment, the upper aperture should be rapidly palpated, and if it is found to be swollen it should be freely scarified with a guarded bistoury guided by the finger; if this fails, tracheotomy or intubation must be performed without delay.

Œdematous laryngitis does not occur as a complication of catarrh in adults, but is usually part of an acute septic inflammation of the throat. Local œdema of part of the larynx—*e.g.* the epiglottis—is seen as a result of perichondritis following a specific fever, especially typhoid, scarlatina, or variola, or is due to secondary infection of a syphilitic, tuberculous, or cancerous lesion.

### Septic Laryngitis.

This is the result of infection by a variety of micro-organisms, especially the streptococci. Usually the entire throat is involved, and the larynx is affected by spread of inflammation from the fauces. The inflammation has many clinical forms; it may be superficial or erysipelatous, membranous, œdematous, suppurative or gangrenous. Though debilitated persons are predisposed, the affection often attacks those in robust health, and for no apparent cause. The onset is sudden, and is followed by all the signs of acute septic intoxication, and the worst cases assume the asthenic type, with dry, brown tongue, muttering delirium, and coma; pneumonia is a frequent complication. Death usually results from septic poisoning and failure of the heart, more rarely from suffocation due to œdema of the larynx, and the prognosis is extremely grave. The first local symptoms are pain and dysphagia, followed by hoarseness, aphonia, and often dyspnœa. The general treatment consists in supporting the strength and maintaining the heart's action by strychnine, digitalis, and the judicious use of alcohol; perchloride of iron in large doses has been recommended. Inoculation with a vaccine made from the organisms found in the patient's throat should be tried when possible, but many cases are too acute to allow time for its preparation; in such a polyvalent antistreptococcic serum may be employed, but is generally disappointing. Local measures consist in the application of cold by means of an ice-bag, leeches to the neck, and scarification of the larynx if œdema be present. If there is definite obstruction, tracheotomy must be performed, and low down, for subglottic swelling may extend down the trachea; but these patients are very ill, and stand the operation badly. The worst cases of septic pharyngo-laryngitis die poisoned within thirty-six hours of the onset of the disease.

